# Skin Carotenoids in Public Health and Nutricosmetics: The Emerging Roles and Applications of the UV Radiation-Absorbing Colourless Carotenoids Phytoene and Phytofluene

**DOI:** 10.3390/nu11051093

**Published:** 2019-05-16

**Authors:** Antonio J. Meléndez-Martínez, Carla M. Stinco, Paula Mapelli-Brahm

**Affiliations:** Food Colour & Quality Laboratory, Area of Nutrition & Food Science, Universidad de Sevilla, 41012 Seville, Spain; cstinco@us.es (C.M.S.); pmapelli@us.es (P.M.-B.)

**Keywords:** colourless carotenoids, cosmeceuticals, functional foods, nutraceuticals, nutricosmetics, photoprotection, phytoene, phytofluene, public health

## Abstract

In this work, the importance of dietary carotenoids in skin health and appearance is comprehensively reviewed and discussed. References are made to their applications in health-promoting and nutricosmetic products and the important public health implications that can be derived. Attention is focused on the colourless UV radiation (UVR)-absorbing dietary carotenoids phytoene and phytofluene, which are attracting increased interest in food science and technology, nutrition, health and cosmetics. These compounds are major dietary carotenoids, readily bioavailable, and have been shown to be involved in several health-promoting actions, as pinpointed in recent reviews. The growing evidence that these unique UVR-absorbing carotenoids with distinctive structures, properties (light absorption, susceptibility to oxidation, rigidity, tendency to aggregation, or even fluorescence, in the case of phytofluene) and activities can be beneficial in these contexts is highlighted. Additionally, the recommendation that the levels of these carotenoids are considered in properly assessing skin carotenoid status is made.

## 1. Introduction

Epithelial tissues like those that form the skin are essential to protect the human body from diverse aggressions. The skin is the largest organ of the human body and consists of several layers and structures. It is an essential physical barrier that protects us from the external environment (radiation, xenobiotics, microorganisms, etc.) but also intervenes in essential processes. Examples are thermoregulation, metabolism, the homeostasis of fluids, sensing, or the production of vitamin D and other important compounds [1,2,3,4,5]. 

Given its importance at different levels, damage to the skin lead to disorders of different natures that can eventually lead to diseases, for instance those derived from infections or even skin cancers, with consequent negative effects in terms of well-being and associated healthcare costs. Skin appearance is also important for individuals at various levels, since some attributes (pigmentation, colour uniformity, wrinkling, elasticity, etc.) have a significant influence on attractiveness judgements, and skin signalling has been associated to aspects ranging from mating choices to socioeconomic status [6,7,8,9,10]. 

Within this context, protection of the skin against sunlight and other sources of radiation (for example tanning lamps) is of great relevance, as excessive exposure in particular to UV radiation (UVR) can lead to photosensitivity, sunburn, photoaging, immunosuppresive effects, or even development of skin cancer, disorders with associated negative health and aesthetic outcomes [11]. 

The role of nutrition in skin health and appearance is undeniable and long-known. In this context, several terms are commonly used and sometimes overlap, such as cosmetics, cosmeceuticals, nutricosmetics, functional foods, or nutraceuticals. Although there is no broad standard consensus definition, the term “nutricosmetics” is associated with the oral consumption of products containing food components (e.g., vitamins, peptides, polysaccharides, polyphenols, coenzyme Q10, polyunsaturated fatty acids, and carotenoids) for cosmetic purposes. Thus, the term is clearly linked to concepts like ‘beauty pills’, ‘beauty from within’, ‘beauty foods’, ‘nutraceuticals for skin care’, or ‘oral cosmetics’ [5,12,13,14].

Carotenoids are natural dietary products ingested from foods, as additives, or from supplements, among other products [15,16]. Some carotenoids can be consistently found in human plasma, milk, and various tissues, including the skin, with major carotenoids usually being lutein, zeaxanthin, β-cryptoxanthin, α-carotene, β-carotene, lycopene, phytoene, and phytofluene [17]. Apart from their role as natural colourants, carotenoids have been important in nutrition because some of them function as provitamin A, and evidence has accumulated over the last 30 years that carotenoids may contribute to decreasing the risk of developing various non-communicable diseases including several types of cancers, cardiovascular disease, and skin or eye disorders, among others [18,19,20,21]. This is the main source of their indisputable value in the context of functional foods, nutraceuticals and related products.

The UVR-absorbing colourless carotenoids phytoene and phytofluene are rarities in the carotenoid family and have been largely ignored in studies dealing with food science and technology, nutrition, public health, and cosmetics. This is surprising since recent comprehensive reviews have indicated that they are major dietary carotenoids (found in widely consumed products, including tomatoes, carrots, citrus, and derivatives), readily bioavailable (they are present as major carotenoids in plasma, human milk, skin and other tissues) and involved in several health-promoting actions, as revealed by various studies. Notably, evidence is accumulating that they could be involved in the health benefits traditionally associated to lycopene [22,23,24]. Being the unique major dietary carotenoids absorbing maximally in the UV region and possessing other distinctive characteristics within the carotenoid family, research, and applications in the use of these carotenoids for the promotion of health and cosmetics is a live and expanding area recently featuring in the carotenoid field. 

## 2. Structure and Functions of the Skin

Epithelial tissues, including the skin, are essential for survival since they protect from physical, chemical, and microbial damage and intervening in homeostasis [3].

### 2.1. Skin Structure

The skin is the largest organ of the human body with a surface area of 1.5–2 m^2^ and accounting for about 15% of the total body weight of an adult. It is composed of epidermis, dermis, subcutaneous fat and structures like hair follicles, as well as sweat and sebaceous glands. The different layers that are distinguished in the epidermis are characterized by the state of differentiation of keratinocytes, the most numerous cells in this skin layer. The outermost epidermis layer is the stratum corneum, with a thickness of 10 μm to 30 μm. The cells in this external layer are called corneocytes and lack nuclei and cytoplasmic organelles. Below the stratum corneum there are keratinocytes (~95% of epidermal cells), melanocytes, Langerhans cells and Merkel cells [1,4,5]. 

The dermis is made up of ~60% water and has a thickness ranging from 0.5–5 mm depending on the location. In it, two main layers are distinguished: the papillary dermis (rich in blood vessels and nerve endings); and the reticular dermis, the main part of the dermis that is in contact with the subcutis. The main dermal cells are fibroblasts, mast cells, plasma cells, lymphocytes, dermal dendritic cells and histiocytes. The dermis also contains interstitial materials, including collagen fibres (~70–90% of the dermis dry weight), elastic tissue or ground substance (that together form part of the so-called extracellular matrix proteins complex) as well as sweat pores and hair follicles. It is estimated that in the dermis there are 1.5–6 million sweat glands, which are of two types, namely eccrine (from which most thermoregulatory sweating and sweat fluid comes from) and apocrine. Within the dermis, different types of mechanoreceptors can also be found [1,4,5]. 

The hypodermis or subcutis is the most internal layer of the skin and contains cells known as lipocytes. It is estimated that almost 80% of all body fat is deposited in the subcutis (in non-obese individuals) [1,4,5].

### 2.2. Skin Functions

The skin is an essential physical barrier that protects the body from the external environment (radiation, xenobiotics, microorganisms, etc.), the epidermis being fundamental for this function. The dermis confers mechanical strength and elasticity to the skin and the subcutis acts as insulation and mechanical protection and contributes to thermoregulation [1,4,5] 

**Figure 1:**
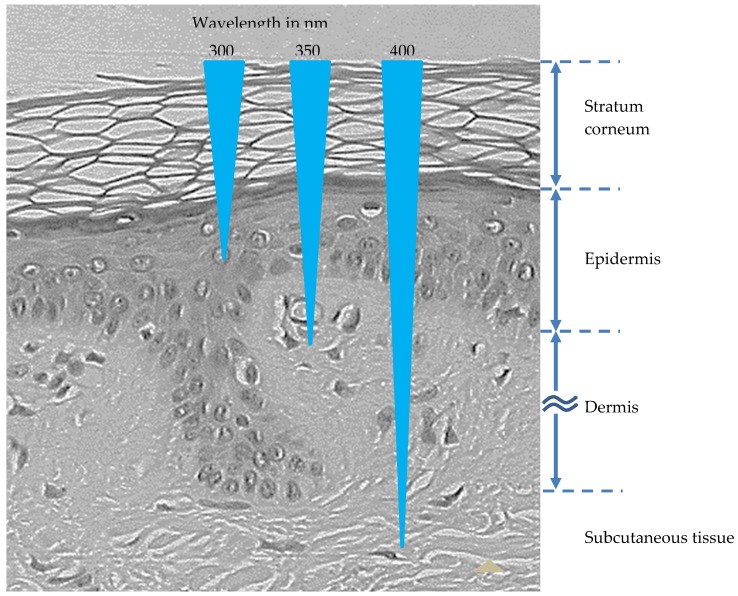
Skin penetration depth of different wavelenghts.

In relation to the protection against radiation, it is to be noted that the degree of light penetration in the skin is wavelength-dependent. Thus, the longer the wavelength (and the lower the energy of the radiation), the deeper is the penetration. Long wave UVA (320–400 nm) can penetrate the whole dermis but the more energetic medium wave UVB (280–320 nm) mainly reaches only as deep as the epidermis. The shortwave UVC (200–280 nm) is absorbed by the ozone layer and does not reach the Earth’s surface. Visible radiation (approximately 400–770 nm) penetrates into subcutaneous tissue. [5,11,25,26] (Figure 1). 

Protection against UV light is of great importance to prevent lesions of different gravity, some of which may lead to an undesirable appearance. For instance, excessive exposure to sunlight or artificial sources of UV light, a situation commonly associated with tanning, lead to adverse effects. These can be categorized into different disorders, including sunburn, immunosuppression, photocarcinogenesis, and photoaging. The latter is characterized by collagen proteolysis that results in signs such as wrinkles. Other signs of photoaging are teleangiectasia (dilated and deformed microvasculature) or hyper-pigmentation [11], all of them undesirable signs from an aesthetic point of view.

Concerning the barrier function of the skin, the stratum corneum has a prominent role as a water-proof, relatively impermeable barrier that is key in preventing the entry of diverse xenobiotics [2] However, many compounds can absorb through the stratum corneum, from which derives the existence of a wide variety of pharmaceutical and cosmetic products intended for topical application [27,28,29,30].

The skin also play key roles in vital processes such as thermoregulation, metabolism and the homeostasis of fluids. In relation to the latter, the skin, particularly the water-impermeable stratum corneum, helps reduce water loss and, therefore, dehydration. The skin also contributes to innate and adaptive immunity. For instance, substances produced in keratinocytes or derived from the sweat can exhibit antimicrobial activity against a wide variety of bacteria, viruses, and fungi. Additionally, the skin’s Langerhans cells act in the immune response against threats. Regarding the role of skin in the regulation of body temperature, the regulation of body temperature and heat loss is effected by sweating and the vasodilatation and vasoconstriction of vascular plexi. Dissipation of excess heat occurs mostly through the skin, by means of the production of sweat from plasma and the subsequent heat transfer to the environment (perspiration) [1,2].

The skin is also important for sensory perception due to the presence of abundant free nerve endings and end-corpuscles. Thus, different kinds of mechanoreceptors (including Merkel’s disks, Meissner’s corpuscles, Ruffini’s endings, Pacinian corpuscles and, in some locations, Krause’s end bulbs) are present [31,32,33]. 

Skin is important for the synthesis of vitamin D and, therefore, has a role in bone synthesis, calcium metabolism and other processes associated with the vitamin. More specifically, the epidermis is considered the major source of vitamin D for humans, as ultraviolet radiation (specifically UVB) can lead to the conversion of 7-dehydrocholesterol to vitamin D. Furthermore, the keratinocytes contain the enzymes required to further convert the vitamin into 1,25 dihydroxyvitamin D, its active form. The layer of subcutaneous fat is important to provide insulation and for mechanical protection, acting as a cushion. Finally, this layer has a role as an energy reserve and in the secretion of hormones [1,4,5,34].

**Figure 2:**
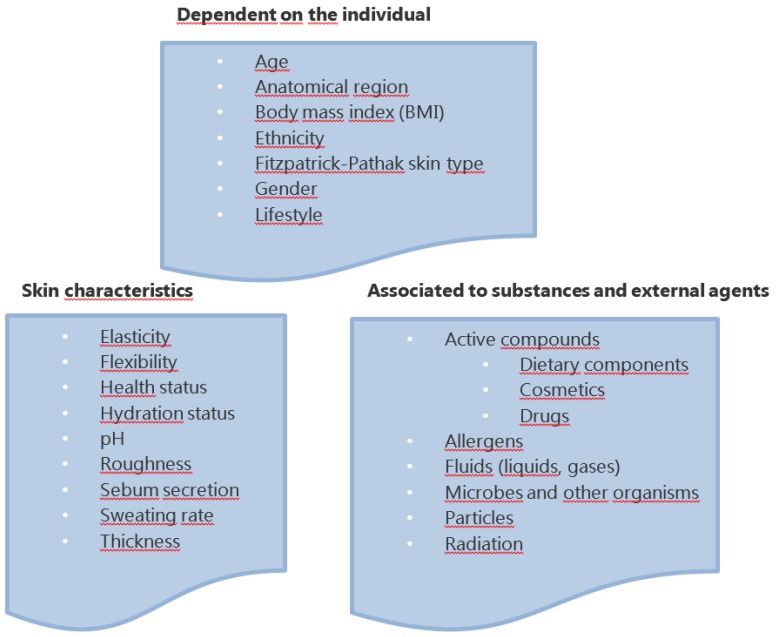
Factors affecting skin properties. Adapted from Dąbrowska et al. [4].

The properties of the skin, related to its functions and individual quality of life, are dependent on manifold factors of varying nature, such as the ones summarized in Figure 2 [4]. Most of the factors related to the individual are congenital. In this regard, it is considered that some factors, notably ethnicity and gender, influence the skin’s properties to a lesser degree. The Fitzpatrick–Pathak skin type, a classification of six different types of skin based on their response to sun exposure (Table 1), has a marked impact on protection against radiation and photoaging, which features reduced hydration and elasticity. Factors dependent on the skin characteristics can vary markedly depending on the body site considered. Additionally, the presence or penetration of different substances may have a marked effect on the skin properties. Indeed, the cosmetic and pharmaceutical industries have made use of the skin’s capacity to absorb such substances [4].

## 3. Skin Disorders

It is estimated that over one thousand disorders involving the skin have been described (infections, drug reactions, psoriasis, eczema, urticaria, acne vulgaris, pityriasis rubra pilaris, Darier’s disease, ichthyosis, lichen ruber, and different skin cancers, including basal cell carcinoma, squamous cell carcinoma, or melanoma, etc.) and that 1/5 of all patient referrals to general practitioners involve skin pathologies. From this it can be inferred that skin disorders involve an important economic cost to both the citizens and the healthcare services [1,28].

### 3.1. UV Radiation Types and Consequences of Exposure

UV radiation (UVR) can be categorized into three types as a function of wavelengths, namely UVA (320–400 nm, accounting for ~95% of the UV radiation reaching the earth), UVB (280–320 nm, accounting for ~5%), and UVC (200–280 nm), the latter not reaching the surface of our planet [26]. 

Exposure to UV radiation may lead to the production of reactive oxygen species (ROS), a generic term used to refer to both oxygen radicals and also some non-radical derivatives of oxygen, including the deleterious singlet oxygen, ^1^O_2_. This is generated from O_2_ by an input of energy rearranging the electrons. Due to continuous exposure to light, this non-radical ROS may be easily formed in the skin or even the eye, where they can directly damage lipids, proteins and even DNA [36]. This compound and/or UVA are thought to be able to regulate the expression of a wide variety of genes, including genes intervening in the cell cycle, apoptosis or photoaging (such as matrix metalloproteases or heme oxygenase). UVB can also cause sunburn and lead to the appearance of mutations and skin cancer through direct interaction with DNA [11]. Interestingly, UVA light is not filtered by window glass (unlike UVB light) and it is estimated that half of UVA exposure takes place in the shade [26]. 

Some of the skin’s mechanisms for protection against UVR are increasing the thickness of epidermis; DNA repair mechanisms; programmed cell death; antioxidant enzymes; and skin pigmentation [26]. There is also ample evidence that dietary components, among which carotenoids, may have a protective role in some cases [11,37,38]. UVR can also increase sensitivity to some drugs, e.g., corticoids, and have beneficial effects beyond the induction of vitamin D synthesis. Thus, UVR may be positive for the treatment of skin pathologies, including psoriasis, morphea, scleroderma, vitiligo, or atopic dermatitis [5].

### 3.2. Skin Disorders Associated to UVR

Depending on the intensity and the continuity, exposure to UVR may lead to disorders of different nature and severity, such as photosensitivity disorders, photocarcinogenesis, sunburn, photoaging, and even photoimmune modulation.

#### 3.2.1. Photosensitivity Disorders

One example of photosensitivity disorder is erythropoietic protoporphyria. This is an uncommon inborn haematological disease that leads to increased levels of protoporphyrin in plasma, red globules, skin and faeces. Protoporphyrin is an endogenous photosensitizer. Upon exposure to light can eventually favour the formation of ROS that cause cellular damage and leads to photosensitivity clinical symptoms. Specifically, the patients experience itching, burning, and pain in the skin exposed to sunlight after even only minutes of exposure, followed by edema, erythema, and purpura [11,39,40,41]. Apart from porphyrins, other endogenous compounds (for instance, flavins or amino acids) act as sensitizing molecules [38]. 

The first classification of skin based on the skin response to the sun was based exclusively on the phenotype (hair and eye colour) [42]. However, it was soon realized that it was not fully valid since people with the same hair or eye phenotype had very different responses to the sun. Therefore, Fitzpatrick developed a new classification based on the responses of people to a brief and simple questionnaire, about the response of the skin to sun exposure. Based on this questionnaire six types of skin were eventually defined. The first four types correspond to white skins and were defined by Fitzpatrick [42]. The last two types are made up of skins that do not suffer sunburn and get suntanned after the initial sun exposure and whose colours before the sun exposure are brown (Type V) or black (Type VI) (Table 1). Types V and VI were defined by Fitzpatrick [43] and Pathak [44]. It is considered that the higher the phototype, the higher the melanin levels and the lower the risk of skin cancer [5]. 

#### 3.2.2. Sunburn

Sunburn, or erythema solare, is the acute inflammatory reaction of the skin, originated by an excessive exposure to natural of artificial UV radiation. The effectiveness of UV in causing sunburn is wavelength-dependent, decreasing with longer wavelengths. In this sense it has been estimated that it takes 1000 times more UVA relative to UVB to produce the same erythemal response. Therefore, it is commonly assumed that sunburn is caused by UVB, which induces cytokine-mediated processes and neuroactive and vasoactive mediators in the skin, resulting in inflammatory responses [5]. 

The classical symptom of this disorder is the reddening of the skin and vasodilation of cutaneous blood vessels, although blisters and ablation of the epidermis can appear in more severe episodes. The sunburn episode occurs approximately 4 h after exposure and reaches the peak in the interval 8–24 h. Normally, it disappears in approximately one day, although the persistence of the erythema depends on age and skin type. Thus, it is considered that sunburn lasts longer (up to several days) in older individuals and those with fairer skin. Sunburn cells are keratinocytes undergoing apoptosis [11,25,26]. 

Minimal erythema dose (MED) refers to the minimal dose of UVB required to cause sunburn and is markedly dependent on the skin type. This parameter is used to evaluate the protection factor of sun screens [11].

#### 3.2.3. Photoaging

The structural and functional decline of skin is categorized into two main categories, namely aging due to the passage of time (intrinsic or chronological aging) or to external factors (extrinsic aging). The latter includes skin aging due to chronic exposure to high UVR (or photoaging) and other agents, namely smoking, pollution, sleep deprivation and poor nutrition [45]. Intrinsic and extrinsic aging can be superimposed. However, chronological aging and photoaging display some differences histologically and are sometimes clinically distinguishable. Chronological aging is clinically associated with increased fragility and loss of elasticity, and photoaging with elasticity loss, irregular pigmentation, dryness and wrinkling, signs mostly derived from the degradation of proteins of the extracellular matrix and aberrations in the production of melanin (leading to the appearance of spots or irregular skin tone). Skin aging in general has an impact on manifold characteristics, processes and functions (regenerative capability, pigmentation, thermoregulation, the strength and resiliency conferred by the collagenous extracellular matrix, etc.) converging into increased skin fragility and susceptibility to diseases [2,5]. 

UVA has a prominent role in photoaging. This radiation favours the production of ROS that may eventually cause mutations in the mitochondrial DNA, leading to defects in the cell energy metabolism that result in further inductions of ROS. Additionally, singlet oxygen can also upregulate matrix metalloproteases directly, leading to further damage to the extracellular matrix and the formation of wrinkles and skin sagging [11,46]. Other features of photoaging include pigmented lesions (actinic lentigines or “age spots”, ephelides or “freckles”, and pigmented solar and seborrhoeic keratosis), teleangiectasia (a widening and deformation of the fine capillaries in skin), dryness and inelasticity. Photoaging signs are more common in locations that are more frequently exposed to UVR like the face, neck, forearms, or the back of the hands [2,11]. 

Besides UVR, both near-infrared (NIR, IRA: 760–1440 nm and IRB: 1440–3000 nm) and visible light (400–760 nm) may also be involved in the development of photoaging. More specifically, it is estimated that the solar radiation energy received at the surface of the Earth is divided in this way: 6.8% UV (0.5% UVB, 6.3% UVA), 38.9% VIS, and 54.3% NIR [47]. NIR can generate ROS in the skin and contribute to photoaging [48]. Analogously, visible light may also cause the generation of ROS in the skin [49]. 

#### 3.2.4. Photoimmune Modulation

There is consistent evidence that excessive UVR induces a number of immunological changes to the immune system [11]. Both UVA and UVB have been shown to be able to cause local and systemic immunosuppressive effects. For example, after UVR exposure, the Langerhans cells (epidermal antigen-presenting dendritic cells produced in the bone marrow) undergo functional and morphological changes leading to their depletion. Exposure to UVR may also result in T cell tolerance [1,5,26].

#### 3.2.5. Photocarcinogenesis

Photocarcinogenesis refers to events initiated by the exposure to solar or artificial light that eventually lead to the development of skin cancer [50]. UVB is thought to be absorbed directly by DNA, causing DNA base structural damage, for instance formation of cyclobutane pyrimidine dimers and pyramidine-pyrimidone photodimers, which may eventually cause mutations and cancer. UVA is considered to provoke indirect DNA damage through the generation of ROS resulting in single-strand breaks and in DNA–protein crosslinks. Indeed, DNA absorbs maximally in the range of UV wavelengths from 245–290 nm, implicating UVB as a primary mutagen. Overall, UVR can cause alterations in DNA integrity and homeostasis, and affect genes, including oncogenes and tumour suppressor genes [5,26]. 

## 4. Skin Beauty

From evidence referred to in different sections of this and other works, it is arguable that the skin reflects health-status as well as intrinsic and extrinsic aging. Also, that apart from serving diverse essential functions, the skin is very important in terms of beauty and appeal. Some key attributes of skin appearance (for instance colour, texture, or elasticity) have been long known to be affected by nutritional status, and even by micronutrients or other minor food components of nutritional interest. Thus, vitamin C deficiency can cause various alterations in epithelial tissues, including impaired wound healing or skin fragility, since this vitamin is involved in collagen synthesis and antioxidant protection. Furthermore, it is well-known that retinoic acid (a form of vitamin A) and derivatives modulate the expression of genes involved in cellular differentiation and proliferation at the skin level, hence their many therapeutic and cosmetic applications. Furthermore, increases of dietary intakes of fruits and vegetables may cause perceptible changes in the yellowness of the skin, whilst excessive provitamin A (usually β-carotene) or lycopene may lead to increased skin pigmentation [5,30,51,52,53,54,55].

### 4.1. Colour and Other Parameters Associated to Skin Beauty

#### 4.1.1. Colour

The main skin pigment is melanin. However, there also exist other chromophore-containing molecules that contribute to skin colouration, including oxyhemoglobin, deoxyhemoglobin, and carotenoids [26]. The distribution of carotenoids across the different skin layers and anatomical regions is not uniform. The highest levels are usually found in the stratum corneum especially close to the skin surface [56,57]. Oxyhemoglobin and deoxyhemoglobin are especially important in the papillary dermis, which is rich in blood vessels [1]. Melanin is a term used to refer to a group of natural pigments that also confer colour to the hair. There are two types of melanin, namely, eumelanin (dark brown-black insoluble polymer) and pheomelanin (light red-yellow sulphur-containing polymer). Melanin is synthesized in melanosomes, special organelles within the melanocytes in the basal layer of the epidermis, and then distributed to surrounding keratinocytes. The biosynthesis of melanin (melanogenesis) can be induced by diverse factors (α-melanocyte-stimulating hormone, stem cell factor, endothelin-1, nitric oxide, adrenocorticotropic hormone, prostaglandins, thymidine dinucleotide, or histamine) that eventually lead to the expression of genes codifying for microphthalmia-associated transcription factor (MITF). This is a master regulator of melanogenesis that, among other actions related to melanogenesis, upregulates the melanogenesis enzymes tyrosinase (TYR), tyrosine-related protein-1 (TRP-1), and tyrosine-related protein-2 (TRP-2). Of these three enzymes, TYR is thought to be the only one exclusively necessary for melanogenesis. The amino acid L-tyrosine acts as a substrate for the enzyme and is generally transported into the melanosome by facilitated diffusion. Skin pigmentation is regarded to be one important photoprotective factor as melanin, besides functioning as a broadband UV absorbent, seems to protect against oxidation. Evidence is accumulating that UVR-induced damage and its repair are also signals for the induction of the synthesis of melanin. Many epidemiological studies point to the fact that there is a lower incidence of skin cancer in individuals with darker skin relative to those with fair skin [1,2,10,26,58].

The number of melanocytes across different ethnicities is very similar. Indeed, the differences in skin melanin-related colour are mainly attributed to the size and distribution pattern of melanosomes, their contents in eumelanin and phaeomelanin, and to metabolic and tyrosinase activity within the melanocytes. For example, there is evidence that East Asians have a yellowish skin colour due to a higher proportion of phaeomelanin to eumelanin, and a spherical rather than ellipsoidal arrangement of the clustered pigments. Likewise, other studies indicated that the most fair-skinned ethnical groups (including European, Chinese, and Mexican) have about half the amount of melanin in their epidermis in comparison to the most darkly pigmented groups (including sub-Saharan African and Indian ethnicities) and that the size of melanosomes seems to vary across these groups, so that the largest melanosomes are found in sub-Saharan African individuals, followed in turn by Indian, Mexican, Chinese, and Europeans [9]. 

Interestingly, independently of the ethnicity group, there is evidence that the melanin volume fraction in the epidermis is positively correlated with the mean dose of surface solar UV-radiation received at the geographical location of the group in question [9].

Skin pigmentation and colour homogeneity are well-known to have a significant influence on attractiveness judgements, above all in women, since they can convey information about age, health status and social status, with some cultural differences [6,7,8,9].

#### 4.1.2. Other Skin Aesthetic Parameters 

Physical appeal in general and facial attractiveness in particular, are well-known to be largely associated with health, mate choices, and social status, such that youthful appearance confers advantages, including socioeconomic ones. Thus, there is constant interest in skin rejuvenation, mostly of the face, which is permanently exposed and perceived by others. In this context, there is evidence in the scientific and technical literature that skin structure conducive to a youthful appearance is a very important attribute of attractiveness, along with such others as facial symmetry and proportions [8,10]. Thus, the cosmetic industry aims to fight signs impairing youthful appearance like loss of elasticity, wrinkles and folds, dryness, or pigmentation disorders (spots, uneven colour, sallowness), which are associated with aging in general [2,5,10,59].

### 4.2. Geographic and Ethnic Differences 

Regarding aesthetic preferences, the cosmetic industry must take into account aspects as diverse as differences in preferences across ethnicities or even environmental factors. Thus, it is well-known that many Asians (above all of the east) have preference for fair skin, whereas Caucasians prefer tanned skin, which in both cases is often associated to better health or economic status. Skin tone is an attribute of beauty of current great relevance for some population groups like African American individuals, since there is evidence that skin colouration in individuals of colour has a deep impact in the way they are perceived. Thus, some studies indicate that individuals of colour with lighter skin are perceived more favourably compared to others with darker skins. Additionally, that among African Americans, African American women with lighter skin tones are perceived as more attractive than their darker skinned counterparts [60,61,62,63]. Additionally, the environmental differences across geographical regions (for instance tropical vs. temperate) have an important impact on skin characteristics (colour, photosensitivity, hydration, etc.), so that cosmetics must be conceived, designed, and customized accordingly [64].

## 5. Functional Foods, Nutraceuticals, Cosmetics, Cosmeceuticals, and Nutricosmetics: Definitions and Concepts

The interest of scientists, physicians, pharmacists and other professionals in the impact of nutrition on skin is not new. However, the growing interest of citizens in health-promoting products and the consequent expansion of functional foods and nutraceuticals in different industries experienced in the last 30–40 years have also had an impact on the cosmetic sector. In this context, there is a series of terms, interrelated to a greater or lesser extent, which are commonly used in those industries.

Although there is no consensus definition for the term “functional foods”, at least in Europe, there is a widely accepted working definition stemming from the concerted action “Functional Food Science in Europe” (FUFOSE), as follows:

(1) “A food that beneficially affects one or more target functions in the body beyond adequate nutritional effects in a way that is relevant to either an improved state of health and well-being and/or reduction of risk of disease”; (2) “not a pill, a capsule or any form of dietary supplement”; (3) “consumed as part of a normal food pattern” [65].

Stephen DeFelice (founder of the Foundation for Innovation in Medicine, FIM) is usually credited with the coining of the term “nutraceutical” some 30 years ago. According to him “a nutraceutical is any substance that is a food or part of a food and provides medical or health benefits, including the prevention and treatment of disease. Such products may range from isolated nutrients, dietary supplements and specific diets to genetically engineered designer foods, herbal products, and processed foods such as cereals, soups and beverages” [66]. Although it refers to products at the intersection between foods and drugs (sometimes they are referred to as “pharma-foods”) there is no consensus definition or legal frame for “nutraceuticals” either [67].

According to Regulation (EC) No 1223/2009 of the European Parliament and of the Council of 30 November 2009 on cosmetic products, “‘cosmetic product’ means any substance or mixture intended to be placed in contact with the external parts of the human body (epidermis, hair system, nails, lips and external genital organs) or with the teeth and the mucous membranes of the oral cavity with a view exclusively or mainly to cleaning them, perfuming them, changing their appearance, protecting them, keeping them in good condition or correcting body odours.” [68]. The US Federal Food, Drug, and Cosmetic Act (FD&C Act) defines “cosmetics” as “(1) articles intended to be rubbed, poured, sprinkled, or sprayed on, introduced into, or otherwise applied to the human body or any part thereof for cleansing, beautifying, promoting attractiveness, or altering the appearance, and (2) articles intended for use as a component of any such articles; except that such term shall not include soap" [69]. 

According to Saint-Leger [70], the composite term “cosmeceuticals”, which also lacks consensus definition and legal identity to date, was coined by Mr. R. E. Reed (as President of the Society of Cosmetic Chemists) in 1962. According to this allegedly first definition, a cosmeceutical was a scientifically designed product meeting rigid chemical, physical and medical standards, intended for external application to the human body, which produces useful, desired results and has desirable aesthetic properties [70].

There is no consensus definition of “nutricosmetics” either, although this term is commonly used to refer to products containing food components that are intended for cosmetic purposes and are administered orally. Thus, the term is also associated to concepts like “inside-out approach” “beauty pills’’, “beauty from within”, “beauty foods”, “nutraceuticals for skin care”, or “oral cosmetics” [12,13,14]. Common food components used in these products are vitamins, peptides, polysaccharides, polyphenols, coenzyme Q10, polyunsaturated fatty acids, and carotenoids [5].

## 6. Dietary Carotenoids

Carotenoids are widespread isoprenoid compounds that appeared very early in the history of life on earth. Their structures, physicochemical properties and activities have evolved so that they play key roles in photosynthesis, communication between species through colour signalling, nutrition, and health and other processes [21,71].

### 6.1. Basics on Dietary Carotenoids 

#### 6.1.1. Sources and Intakes

The main dietary sources of carotenoids are fruits and vegetables [15]. However, carotenoids are also present in other components of the diet, like herbs, legumes or cereals [72], algae [73,74], foods of animal origin (egg yolk, mammals’ milk and tissues, seafood) [75,76,77,78,79] additives (colourants) [80,81] and in the form of supplements [16,82].

The diets of humans usually contain ~50 carotenoids, although not all of them are found at detectable levels in humans [17,21]. The normal dietary intakes of major carotenoids in various countries, as recently reviewed, are within the range 0–10 mg/day [21].

#### 6.1.2. Presence in Plasma, Other Biological Fluids and Tissues

Traditionally, it has been considered that the main dietary carotenoids are lutein, zeaxanthin, β-cryptoxanthin, α-carotene, β-carotene and lycopene. These, along with phytoene and phytofluene are usually the major carotenoids in human fluids and tissues [15,17,83,84,85], at typical levels in the range of 0–2 μmol/L (plasma) and 0–1 nmol/g (tissues) [84,86].

#### 6.1.3. Health-Promoting Biological Actions

Provitamin A carotenoids are key in the fight against vitamin A deficiency, a global health problem leading to different manifestations (xerophthalmia, negative effects on growth, compromised immunity, etc.) and that is responsible for large numbers of child mortality [87,88]. In addition, evidence has accumulated over the last 30 years that carotenoids contribute to decreasing the risk of developing non-communicable diseases like several kind of cancers, cardiovascular disease, bone, skin, neurological, metabolic or eye disorders [18,19,54,89,90,91,92,93,94,95,96]. The importance of carotenoids in the context of functional foods, nutraceuticals and related products for health promotion is therefore undeniable.

### 6.2. The “Undercover” Colourless Carotenoids Phytoene and Phytofluene 

Phytoene and phytofluene are carotenoid rarities since they are colourless. They are very important from a biosynthetic point of view because they are precursors of the rest of the carotenoids; hence, they have been thoroughly studied in relation to their biosynthesis. The typical committed step of carotenoid biosynthesis within the isoprenoid route is the condensation of two C_20_ molecules to form (15*Z*)-phytoene, the major geometrical isomer of this carotenoid in most carotenogenic organisms [21,97]. 

Surprisingly, phytoene and phytofluene have been largely ignored in food science and technology, nutrition, health or cosmetics, relative to the other major carotenoids found in human fluids and tissues (lutein, zeaxanthin, β-cryptoxanthin, α-carotene, β-carotene, lycopene). This is probably largely due to a considerable lack of analytical data compared to these latter carotenoids. This in turn is attributable to their lack of colour. Indeed, before the advent of modern detectors attached to liquid chromatography (like diode-arrays allowing the simultaneous monitoring of different wavelengths, or mass spectrometry detectors) their lack of colour made their analysis more difficult, since they absorb mainly UV radiation rather than visible light like virtually all the rest of carotenoids [23]. Currently, there is a fast-expanding interest in these colourless carotenoids because the critical analysis of the literature in recent reviews [22,23,24] has revealed that:

- They are present in widely consumed foods;
- Their intakes and levels in plasma and tissues are comparable (or even superior in some cases) to those of other major dietary carotenoids; and
- They are involved in biological actions that result in health and cosmetic benefits.

#### 6.2.1. Distinctive Chemical Features among Carotenoids 

**Figure 3:**
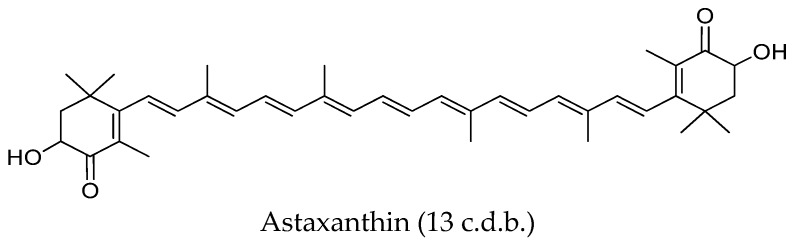

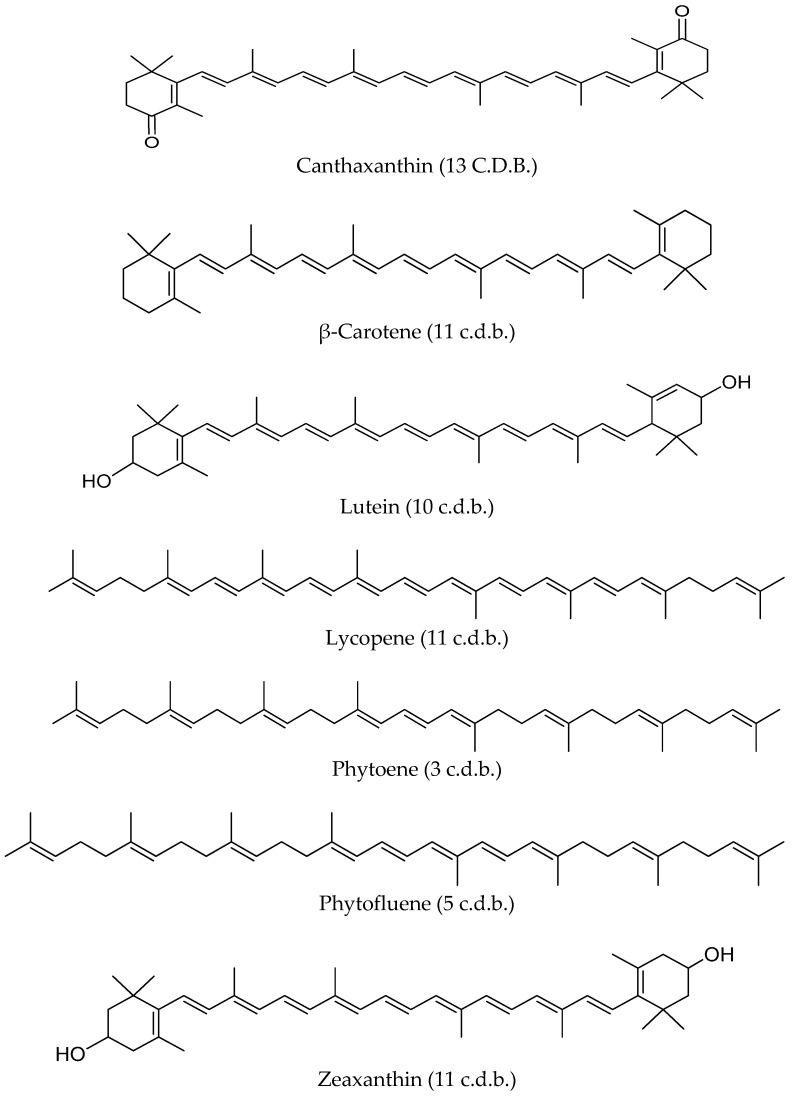
Chemical structures of some carotenoids of interest for skin health and appearance.

The system of conjugated double bonds (c.d.b.), that is, of alternate single and double bonds, is the main structural characteristic of carotenoids. It is the main feature responsible for their physicochemical characteristics (light-absorbing properties, shape or reactivity) and activities [98]. Phytoene and phytofluene are linear hydrocarbons with three and five c.d.b., respectively (Figure 3). Since their polyene chain is markedly shorter than that of other dietary carotenoids (for instance lutein contains 10 c.d.b., zeaxanthin, lycopene and β-carotene 11 c.d.b., and astaxanthin and canthaxanthin 13 c.d.b.) (Figure 3), both colourless carotenoids are expected to exhibit important differences in some physico-chemical properties and biological actions, compared to other carotenoids [23].

##### UV Light Absorption

**Figure 4:**
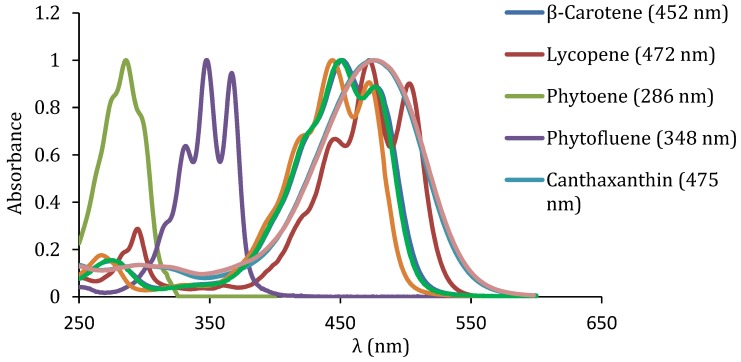
UV–VIS spectra of diverse carotenoids important for photoprotection and cosmetics.

Due to their shorter polyene chain, phytoene and phytofluene absorb maximally in the UV region and are colourless (Table 2). Indeed, it is accepted that 7 c.d.b. are needed for a carotenoid to exhibit appreciable colour [99]. More specifically, phytoene absorbs maximally in the UVB region (280–320) and phytofluene in the UVA region (320–400 nm), unlike virtually the rest of carotenoids in general, including the main carotenoids being studied in relation to skin health and appearance in particular (Table 2) [23]. Their UV–VIS spectra are shown in Figure 4.

The detection of phytofluene can be enhanced by its intense greenish-white fluorescence. Indeed, it is known to fluorescence at around 510 nm when it is excited with near-UV light [101,102].

##### Susceptibility to Oxidation

The characteristic long chromophore of c.d.b. of coloured carotenoids is thought to be closely related to their susceptibility to oxidation and antioxidant/prooxidant properties [98]. Being significantly less unsaturated, the colourless carotenoids could be expected to be more stable towards oxidation under certain conditions. In a recent study, the interaction of phytoene, phytofluene and lycopene with the synthetic oxidizing 2,29-azinobis-(3-ethylbenzothiazoline-6-sulfonic acid) (ABTS) radical cation was assessed by Density Functional Theory and ABTS radical cation decolouration assay strategies. The results suggested that the colourless carotenoids (with 3 and 5 c.d.b., respectively, Figure 3) are not as effective antiradicals as lycopene (11 c.d.b., Figure 3) [103]. Although data on the oxidative stability of phytoene and phytofluene in comparison to other carotenoids in tissues are lacking, there is evidence that they could be more stable in food matrices, for example thermally-treated tomato products [104]

##### Rigidity and Tendency to Aggregation

Their characteristic system of c.d.b. confer carotenoid molecules with rigidity. Since the number of c.d.b. is much lower in phytoene and phytofluene as compared to lycopene for example (another linear carotene), they are expected to adopt shapes notably less rigid relative to most carotenoids in general and lycopene in particular [23,105]. This is expected to have an important impact in terms of their release from the food matrix and incorporation into micelles (“bioaccessibility”). Bioaccessibility is one of the key factors explaining carotenoid bioavailability, since carotenoids must be incorporated into these structures to be taken up by the enterocytes prior to their transfer into the bloodstream [17,106]. In this sense, evidence is accumulating that the bioaccessibility of the colourless carotenoids is markedly higher relative to other carotenoids, probably partly due to their less rigid shape (due to their lower number of c.d.b., but also to their geometrical configuration) and they are less prone to aggregate and form crystals, which are regarded to impair carotenoid release from the food matrix [105,107,108]. 

**Figure 5:**
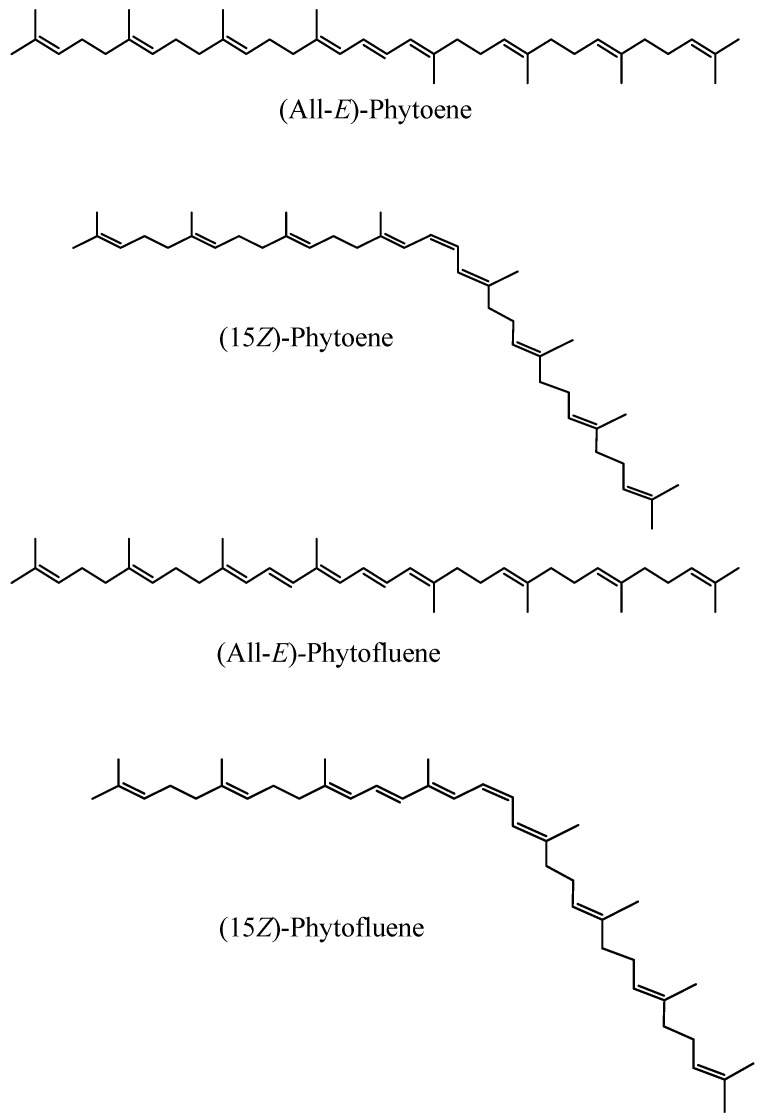
Chemical structures of (all-*E*)- and (15*Z*)-isomers of phytoene and phytofluene.

Carotenoids undergo geometrical isomerization, so all-*trans* (all-*E*) and *cis* (*Z*) isomers can exist. The shapes and sizes of these isomers vary considerably (Figure 5). Thus, the former are linear and rigid and the latter have a bent shape. The geometrical isomerism in carotenoids is thought to have an impact in their solubility, tendency to aggregation, reactivity, bioavailability, or interaction with enzymes, hence the importance of discerning between geometrical isomers [85,105]. Regarding phytoene and phytofluene, it is thought that the (15*Z*)-isomer is usually the major isomer [102]. 

It has been recently shown that both carotenoids are incorporated into mixed micelles much more efficiently than other carotenes, notably lycopene. Unexpectedly, it has also been observed that their micellization efficiency was similar to that of the xanthophyll lutein, observation that challenges the paradigm that xanthophylls have a higher micellization efficiency compared to carotenes. These results have been attributed to the fact that phytoene and phytofluene were mainly present in the form of *Z*-isomers and/or to these carotenes’ higher molecular flexibility, due to their shorter polyene chain [109].

#### 6.2.2. Sources and Intakes

High contents of the colourless carotenoids are found in tomatoes, red grapefruits, watermelon (typically along with lycopene in these three cases), apricot, carrots, and some peppers. Other sources are cantaloupe, banana, melon, oranges, lemon, clementines, avocado, mandarin, nectarine, peach, and exotic fruits (caja, buriti, mamey, marimari, physalis, or gac) [23,24]. The daily dietary intakes of colourless carotenoids was recently assessed in Luxembourg, The percentage intake of phytoene+phytofluene was estimated to be 16% (2.7 mg, especially 2.0 mg for phytoene and 0.7 mg for phytofluene) of total carotenoid intake. Noticeably, the estimated daily intake of lycopene was lower (1.8 mg) than that of phytoene [110].

#### 6.2.3. Presence in Plasma, Other Biological Fluids, and Tissues

Plasma levels of phytoene and phytofluene in the range of 0.04–0.33 μM have been reported [111,112,113] (Table 3). These compounds are also present in breast milk at comparable levels to the other major carotenoids (in the μg/dL range) [114]. Their presence in several human tissues (lung, breast, liver, prostate, cervix, colon, and skin) has been reported, at ng/g levels [115] (Table 3).

#### 6.2.4. Health-Promoting Biological Actions 

Evidence is accumulating that phytoene and phytofluene could be involved in the health benefits traditionally attributed to lycopene, since some reviews suggest that such benefits have usually been observed when tomato products (also containing the colourless carotenoids and other compounds) rather than pure lycopene were used [116,117]. Aside from this, there are studies of different nature (in humans, animals, cell cultures, isolated lipoproteins) indicating that phytoene and phytofluene may be involved, either on their own or together with other compounds, in health-promoting biological actions such as protection against oxidation [118,119,120], inflammation [121,122,123], or anticarcinogenic activity [124,125,126,127,128].

#### 6.2.5. Safety of Phytoene and Phytofluene 

The safe use of phytoene and phytofluene in topical applications is backed by various evidence, namely in vitro cytotoxicity and genotoxicity studies or human in vivo 48-h patch tests and a longer-term Human Repeat Insult Patch Test [29]. The safety of oral formulations can be presumed. On one hand, these compounds are naturally present in many fruits and vegetables. Indeed, estimated daily intakes of phytoene + phytofluene of ~2.70 mg have been proposed for Luxembourg citizens [110]. Furthermore, several phytoene and phytofluene-rich products, notably tomato-derived, have been regarded as safe for human consumption by different competent bodies, including the US FDA or the European Food Safety Authority (EFSA). More especifically, several tomato products have been categorized as GRAS by the FDA, including tomato pulp powder or concentrated tomato lycopene extract [129,130]. Similarly, EFSA considers colourless carotenoid-containing tomato oleoresins safe for human consumption [131].

## 7. Carotenoids in the Skin

### 7.1. Deposition Mechanism 

Regarding the delivery and distribution of carotenoids into the skin layers, there appear to be two main pathways, namely 1) diffusion from the adipose tissue, blood and lymph; and 2) secretion through sweat and/or sebaceous glands onto the skin surface and subsequent penetration [56].

Based on the observation that in untreated skin the larger carotenoid fraction is detected in the external part of the stratum corneum, it has been hypothesized that carotenoids are delivered to that location through secretions by eccrine sweat glands and/or sebaceous glands, similarly to what occurs with vitamin E [57]. In relation to this, it has been observed that, upon consumption of carotenoid-rich products, the increase in the dermal level of carotenoids occurs within 1–3 days, whereas the process of stratum corneum renewal takes 2–3 weeks [132]. According to these authors, the deposition of carotenoids in the skin surface requires their diffusion from the blood, hypodermis and the dermis to the epidermis. Then, the carotenoids would be transported from the blood, the hypodermis and the dermis into the sweat glands, and subsequently to the skin surface with sweat. This explanation is consistent with the fact that the highest levels of skin carotenoids are detected in locations with high numbers of sweat glands [132].

It is hypothesized that the subcutaneous tissue is a storage site for carotenoids and that these are loaded into the keratinocytes which are continuously formed at the basal layer and then migrate to the skin surface, transporting along the carotenoids. In this regard, the time the keratinocytes take to travel from the basal layer to the skin surface (ca. six weeks) matches very well with the time supplementation studies with carotenoids take in producing perceived photoprotection [38].

Finally, it is known that the application of carotenoids topically, results in increased levels in the stratum corneum [56].

### 7.2. Assessment of Skin Carotenoid Levels

To date, the two major strategies to assess dermal carotenoid levels are classical HPLC analysis and non-invasive spectroscopic methods.

#### 7.2.1. HPLC Analysis

The HPLC analysis of skin requires the obtaining of biopsies and a suitable extraction method. Apart from the volunteer’s discomfort, this is a time-consuming approach that may also lead to the oxidative loss of carotenoids. Therefore, it is not suitable for high throughput analyses or the analysis of kinetics of carotenoid levels changes in human skin. Some methodologies are found in recent studies for validation of resonance Raman spectroscopy approaches [133,134].

#### 7.2.2. Non-Invasive Spectroscopic Methods

Resonance Raman spectroscopy (RRS) and light reflection spectroscopy (LRS), including the assessment of colour parameters from reflectance measurement are optical methods used for the rapid, almost instantaneous evaluation of carotenoid levels in skin. 

They are non-invasive, so that the performance of biopsies is not required, with obvious advantages. Given that skin carotenoids are a biomarker of fruits and vegetables intake, the use of these techniques is very useful to screen large population sets for nutritional and epidemiological purposes in the context of public health. To avoid interferences from other sources of colour in skin, the measurements should be made in areas with minimal melanin content, for example the heel of the palm, the tip of a finger, or the heel of the foot. RRS is a more widely used and thoroughly validated method. LRS is an emerging alternative with some advantages, including higher simplicity, portability and the possibility of quantifying the major tissue chromophores and taking them into account to estimate skin carotenoid levels, made possible through detecting reflection over a wider spectral range, from the near UV to the near IR regions [135]. 

A major drawback of spectroscopic approaches relative to HPLC analyses is that the former do not take into account the levels of the colourless carotenoids. Thus, in RRS carotenoids are usually excited in the typical maximal absorption band of coloured carotenoids in the blue region of the visible spectrum. LRS results in the calculation of a score for coloured carotenoid compounds absorbing in the 460–520 nm range [135].

##### Light Reflection Spectroscopy (LRS)

The LRS methodology, which was first applied to the assessment of dermal carotenoids ~20 years ago [136,137] is more simple and requires much smaller devices, some of which are portable. Thus, even a low-power white lamp can be used as a light source and the carotenoid levels are derived from the reflection spectra. Currently, the LRS leads to the calculation of a composite score for all carotenoids absorbing in the 460–520 nm range. Some limitations that this methodology needs to overcome are the interference of the optical properties of the skin (related to autofluorescence, skin hydration, and sweat and sebum production), including those derived from other coloured molecules, namely melanin, haemoglobin, or oxyhemoglobin [138]. Although the technique is not as widely validated as RRS [56,135,139] its use is becoming more popular [140,141].

Another approach based on reflectance measurement consists in measuring colour parameters [135] Indeed, correlations between skin colour parameters and carotenoid levels have been recently reported [142,143]. The assessment of carotenoids from colour parameters derived from reflection spectra has also been widely used in food science and technology to estimate concentrations of a wide diversity of carotenoids [144,145,146,147], notably such as those occurring in industrially-processed orange juices, with different chromophores and functional groups [148].

##### Resonance Raman Spectroscopy (RRS)

Details about the basics of the technique are provided elsewhere [135,139]. RRS methods are based on the use of laser or LED light sources of narrow spectrum’ and detects the characteristic vibrational/rotational energy levels of a molecule. The characteristic polyene chain of carotenoids makes them well suited for detection by this method. They are characterized by three intense Stokes lines at 1005 cm^−1^ (due to the rocking motion of the methyl group), 1156 cm^−1^ (owed to a carbon-carbon single-bond stretch vibration of the polyene chain) and 1523 cm^−1^ (carbon–carbon double-bond stretch vibration of the conjugated polyene) [56]. The Raman line intensities have been shown to have good linear correlation with the physiological skin carotenoid levels [134,135,139].

RSS has become a valuable tool for different types of studies, for instance the use of dermal carotenoids as nutritional biomarkers in diverse groups [133,149,150,151] or the study of associations between carotenoid status and several conditions [152]. 

#### 7.2.3. Major Skin Carotenoids 

It is accepted that, in general, carotenoid levels in the skin reflect those present in plasma [153]. Major human circulating carotenoids (lutein, zeaxanthin, β-cryptoxanthin, β-carotene, lycopene, phytoene, and phytofluene) have been detected in the skin, usually in the range of 0–10 nmol/g wet tissue (Table 4), although higher concentrations are detected in case of dietary supplementation or in carotenodermia [11,113,154]. The presence of other less common carotenoids (α-cryptoxanthin and anhydrolutein) has also been reported [155]. Interestingly, carotenoid esters have been found in the skin, unlike in other tissues. Indeed, it is well-known that esterified xanthophylls are hydrolyzed during digestion and absorbed as free xanthophylls [17,156,157]. Specifically, up to eighteen esters (including both mono- and di-esters) of lutein, zeaxanthin, 2’,3’-anhydrolutein, α-cryptoxanthin, and β-cryptoxanthin have been described in human skin, although at levels considerably lower (pmol/g) than those of free carotenoids [155].

#### 7.2.4. Factors Affecting Skin Carotenoid Levels

Obviously, there are important individual differences in the skin carotenoid levels, as a result of the manifold factors affecting carotenoid bioavailability, from dietary patterns to lifestyle and genotypic factors [17,158,159]. The skin levels of antioxidants are known to depend not only on the diet, but also on skin location or type, individual characteristics (gender, age), health status, or stress factors, both environmental and derived from lifestyle [56,132].

##### Diet

Dermal carotenoid levels can be increased in a relatively short time by dietary means. There is ample evidence that not only supplements, but also fruits and vegetables increase carotenoid skin levels [160,161,162] and that this may have a noticeable impact in terms of skin colour [55,163]. Occasionally, excessive carotenoid intake leads to carotenodermia, an apparently innocuous phenomenon featuring noticeable orange pigmentation of the skin as a result of elevated carotenoid deposition mainly in the stratum corneum, sweat, and sebum. The condition can be due to high carotenoid intake (normally of β-carotene), metabolic disorders, or familial carotenemia. It is believed that it appears when the serum carotenoid levels are in the range of 2.5 mg/L. Following the cessation of excessive carotenoid ingestion, the possible altered biochemical parameters normalize within several weeks. A similar condition due to very high intakes of lycopene has been sometimes termed lycopenemia [52,53,164].

##### Skin Location

Concerning carotenoids’ distribution in different skin areas, there are marked differences, such that it has been reported that the highest levels can be detected in the forehead and the palm of the hands and lower concentrations are found in the back of the hands, inside the arm or in the dorsal area [57,136]. Additionally, the distribution of carotenoids across skin layers is not homogeneous. It appears that the maximum levels are found close to the skin surface (depth of ~4–8 μm), and levels decrease at least up to the depth of 30 μm [56,57]. 

##### Individual Characteristics

There is evidence that gender and body mass index (BMI) could have an impact on dermal carotenoid status. Thus, a recent study observed higher values in women and volunteers with BMI below 30. Age did not seem to have an effect, except in the case of lycopene [165]. 

##### Stress Factors

External stress factors (e.g., radiation) or derived from lifestyle (smoking) or disease (cold, psoriasis or cancer) have been shown to have a negative impact on carotenoid levels in the skin. 

Thus, in a classical study, controlled exposure to sunlight was shown to lead to noticeable decreases of circulating and skin carotenoid levels in human volunteers [166]. Exposure to visible blue-violet and infra-red light has also been shown to lead to the decrease of skin carotenoids, likely through the production of ROS [167,168,169]. In an interesting study the topical administration of β-carotene resulted in a protective effect [169].

As an example of lifestyle factors, it has been observed that, overall, smokers had lower levels of skin carotenoids relative to non-smokers [165]. 

Psoriasis is an inflammatory disease that affects the skin but can also have impacts at the nutritional level. In a cross-sectional study, it was concluded that patients with psoriasis had lower dermal carotenoid levels as assessed in the palm of the hand. However, the carotenoid levels were not significantly associated with the severity of the disorder [170].

A recent study involving 102 breast cancer patients evaluated possible associations between anxiety and skin carotenoid levels (assessed non-invasively as skin carotenoid score, SCS) as a measure of oxidative stress, since chronic stress has been associated with tumour progression, higher recurrence rates and increased risk of metastasis of this malignancy. The results indicated that higher levels of skin carotenoids were associated with decreased severity of anxiety and other miscellaneous parameters (lower body max index (BMI), higher intakes of fruits and vegetables, Hispanic race, lower educational status, and non-smoking) [171].

#### 7.2.5. Kinetic Aspects

In a very interesting work Darvin et al. [132] evaluated the changes in the levels of β-carotene and lycopene (assessed by resonance Raman spectroscopy) in human skin over one year in relation to diet and exposure to stress factors as assessed via questionnaires. The skin carotenoid levels in all volunteers were noticed to be higher in summer and autumn, which was mainly attributed to the increased intake of fruits and vegetables and decrease occurrence of illnesses. Overall, they concluded that large intakes of fruits and vegetables clearly enhanced the levels of these two carotenoids in the skin, while stress factors (radiation, fatigue, illness, smoking, or alcohol intake) decreased them, probably in relation to the production of reactive oxygen species. The kinetics of the observed rises in carotenoid levels owed to fruit and vegetable intakes, and the corresponding decreases seemed due to stress factors behave differently. The decreases took place relatively quickly (over the course of 2h), while the subsequent recovery usually took up to three days to level. Both increases and decreases in carotenoid levels took place much faster than the subsequent return to basal levels [132]. 

Another study showed that skin carotenoid levels rose quickly with increased carotenoid intakes, and that such rises were useful to evaluate compliance in intervention studies as soon as two weeks after start of the dietary intervention [161]. Circulating carotenoid levels returned to baseline after three weeks, which is in good agreement with depletion studies showing decreases in blood carotenoids within 2–3 weeks. In the latter, the dermal carotenoid levels did not return to baseline until volunteers returned to their usual diets for ca. one month. The authors argued that this could be due to the fact that skin acts as a storage tissue for carotenoids. This argument agrees with the findings of other authors after a supplementation trial suggesting that the delayed drop in the skin carotenoid levels relative to those in blood may indicate a peripheral buffer function of the skin for carotenoids [172]. The study by Jahns et al. [161] concluded that skin carotenoid levels assessed by RRS was a valid biomarker of change in skin carotenoid status through fruit and vegetable consumption at recommended intake levels in the US, but with a longer half-life relative to blood carotenoids and the additional advantage that the methodology is non-invasive [161].

## 8. Carotenoids and Photoprotection

### 8.1. Carotenoids and Protection Against Light in Diverse Organisms and Locations

Carotenoids are used in photosynthesis to aid in collecting light of certain wavelengths and protecting from light-derived damage in photosynthetic organisms, including cyanobacteria, considered to be the most ancient oxygenic photosynthetic organisms and the origin of plant chloroplasts [173]. Other organisms that appeared on earth later, also used them in liaison with light: for instance, they play essential roles (quenching of excited chlorophyll or singlet oxygen, light harvesting, assembly of protein-pigment complexes, among others) in photosynthetic tissues in other organisms. Additionally, they are important for vision, including in functions beyond the role of some carotenoids as precursors of retinal for the visual cycle. For instance, some birds have carotenoid-containing oil droplets, through which light passes before reaching the photoreceptors in the retina. This seems to be a means of filtering shorter (more energetic) visible wavelengths and enhancing the sensitivity and perception of colours [174]. 

Strikingly, the human macula lutea, the yellow spot in the central part of the retina (with a diameter of 5–6 mm) has the highest local concentration of carotenoids in humans. This area contains the fovea (with a diameter of ~1.5 mm), which is the area with the highest visual acuity. More specifically, only lutein and zeaxanthin (in the form of two stereoisomers (3*R*,3’*R*)-zeaxanthin and (3*R*,3’*S*)-zeaxanthin, the latter of non-dietary origin and also referred to as *meso*-zeaxanthin) of the circulating human carotenoids are found in the macula lutea [175]. *Meso*-zeaxanthin is thought to be formed in vivo from lutein [176]. This selective accumulation is due to the existence of specific transporters for these two carotenoids, such as a Pi isoform of glutathione S-transferase (GSTP1) (which has been shown to be a zeaxanthin-binding protein in the human macula) [177] or a member of the steroidogenic acute regulatory domain (StARD) (which has been demonstrated to bind lutein in the primate retina) [178]. The presence of such high amounts of just two specific carotenoids in this area of the retina, where light reaches with high intensity is unlikely to be fortuitous. Indeed, the macular carotenoids are thought to be important to reduce the risk of developing age-related macular degeneration, maybe, at least in part, by absorbing blue light and, thus, protecting the photoreceptor cell layer from light-induced damage that could be initiated by the formation of reactive oxygen species during a photosensitized reaction [179]. Thus, carotenoids are important in the protection of different organisms against damage caused by light. Consequently, it is reasonable to expect that they can also have such a role in the skin, which is exposed to sunlight on a daily basis. 

### 8.2. Sunscreens vs. Dietary Approaches

When it comes to providing protection from sunlight, sunscreens are usually the method of choice. However, dietary approaches may also be used, which entails additional benefits. 

Photoprotection based on dietary components in terms of sun protection factor is regarded to be markedly lower than that achievable by using topical sunscreens. Additionally, whilst the protection conferred by the latter is virtually instantaneous, the intervention studies carried out with coloured carotenoids indicate that it takes 7–10 weeks until the protection against sunburn becomes significant. However, it is argued that the presence of dietary antioxidants and other compounds from the diet is a good and natural strategy to endow the skin with basal defences against photodamage and other aggressions that can also affect appearance [54]. β-carotene has been shown to protect against photodamage caused by visible and infra-red radiations and may be an effective antioxidant in sunscreens [49,169]. However, carotenoids are very unstable compounds, and strategies to overcome this fact are needed for the meaningful formulation of carotenoid-containing products. In this context, as a result of a study in which the topical photoprotection of β-carotene was assessed it was recommended that products intended for topical use should consist of a mixture of antioxidants reflecting those present in the skin rather than single antioxidants [169]. 

### 8.3. Mechanisms of Skin Protection by Carotenoids 

Apart from possible cosmetic benefits, another great advantage of increasing the intake of carotenoids and other dietary components for photoprotection is that their levels in plasma and other tissues can also be enhanced, with consequent health-promoting benefits. There is ample evidence indicating that carotenoids contribute to decreasing the risk of developing diseases, including cancer, cardiovascular, and metabolic diseases [19,21,22,23,24].

Goralczyk and Wertz [11] identified some major mechanisms, among which inhibition of lipid peroxidation, inhibition of UVA-induced expression of heme oxygenase 1, prevention of mitochondrial DNA mutations, inhibition of metalloproteases and photoimmune modulation. Recent experiments in hairless mice subjected to UVB radiation indicated that tangerine tomato carotenoids may exert beneficial effects by attenuating DNA-damage and inflammation, with interesting differences between males and females [180]. Noticeably, new possible mechanisms are hypothesized thanks to the use of omics technologies for the study of changes in gene expressions associated to exposure to carotenoids. In any case, attribution of the effect to a single mechanism does not seem possible, and there may be connections among them in some cases [11]. 

Further, provitamin A carotenoids are beneficial for the skin through the production of retinoic acid, which plays important roles at this level, as do other retinoids. They are thought to intervene in processes including keratinocyte proliferation, epidermal differentiation and keratinisation, reduction of inflammation or oxidation, or even the enhancement of the penetration of agents administered topically, among many others. Thus, retinoids are applied for different purposes, for example improving wound healing, preventing skin aging or the treatment of acne, psoriasis, or other skin conditions [28,181]. However, this topic will not be discussed in detail in this review.

#### 8.3.1. Inhibition of Lipid Peroxidation

The peroxidation of diverse lipids (importantly free and esterified cholesterol, and polyunsaturated fatty acids) by enzymatic or non-enzymatic means leads to the formation of various products that act as redox signalling molecules. They may have negative effects at the level of structures like membranes or molecules (proteins or DNA bases) that eventually lead to disease states [182]. 

It is well-known that carotenoids intervene by quenching singlet oxygen or scavenging free radicals, both in solution and in other systems such as membranes or cells, although they can also interact with other antioxidant and non-antioxidant compounds or even act as pro-oxidants under certain conditions [19,46,183,184]. Discussion about the protection by carotenoid-containing products against lipid oxidation is found elsewhere [185]. 

Interestingly, there is evidence that carotenoids may also be prooxidants depending on factors including their concentration, oxygen tension, exposure to radiation, or interaction with other compounds. The resulting prooxidant effects could be harmful or beneficial [186,187,188].

#### 8.3.2. Inhibition of UVA-Induced Expression of Heme Oxygenase 1

Heme oxygenase-1 (HO1) is a ubiquitous enzyme that catalyses the first reaction in heme degradation, which eventually leads to the formation of CO, biliverdin, and Fe^2+^. The enzyme is involved many processes related to the regulation of cell proliferation, differentiation and apoptosis [189]. The expression of the gene codifying the enzyme is inducible. As an example, it is activated via singlet oxygen within the first hours after the exposure of skin fibroblasts to UVA. Some studies in cell cultures involving mainly β-carotene in different formulations suggest that this could be a possible mechanism of carotenoid protection [11].

#### 8.3.3. Prevention of Mitochondrial DNA Mutations

Mutations (typically a ~5000 base pair deletion usually termed “common mutation”) at the level of the mitochondrial DNA, are thought to be involved in negative effects including skin chronological and UV-induced aging and carcinogenesis. Indeed, these mutations have been shown to be increased in aged skin. There is evidence from cell culture studies that β-carotene (and/or some oxidative metabolites) could protect fibroblasts by reducing the occurrence of such mutations [11].

#### 8.3.4. Metalloprotease Inhibition

Matrix metalloprotease (MMPs) encoding genes are regarded to be among the most important genes involved in photoaging, whose expression is induced by singlet oxygen. MMPs are members of a family of enzymes (collagenases, gelatinases, stromelysins, some elastases, and aggrecanases) that catalyze the normal degradation of extracellular matrix (ECM) macromolecules including collagens, proteoglycans (aggrecan, decorin, biglycan, fibromodulin, and versican) and accessory ECM proteins like fibronectin [190]. 

UVR can activate cell-surface growth factors and cytokine receptors by a ligand-independent mechanism. This induces several signalling pathways that result in the stimulation of the transcription factor AP-1, which upregulates genes of several members of the matrix metalloprotease (MMP) family. This increases the proteolysis of proteins of the extracellular matrix, mostly collagen, but also fibronectin, proteoglycans, and elastin, and results in skin elastosis and wrinkling. MMPs are also thought to be important in photocarcinogenesis since they intervene in cell growth, angiogenesis and metastasis. If the high exposure to UVR is chronic, other signs, such as dilated and twisted microvasculature (teleangiectasia) or hyper-pigmentation may appear, these being clinical features of photoaging [5,11].

The use of omics technologies for the overall assessment of changes in the expression of genes in whole genomes is expanding the knowledge of possible biological actions of carotenoids and mechanistic aspects. In this sense, the results of a classical study about overall changes of gene expressions due to treatment of keratinocytes with β-carotene (at physiological dose levels, namely 0.5, 1.5, and 3.0 μmol/L) prior to UVA exposure indicated that carotenoids can act in these cells by modulating the expression of genes related to multiple pathways. More specifically, it was concluded that of the 568 genes whose expression was regulated by UV, the carotene reduced the effect of radiation for 143 and enhanced it for 180 [191]. In this sense, it would not be surprising that carotenoids protect the skin by several of the major mechanisms pinpointed. Thus, a recent study has shown that supplementation with two products containing different carotenoids (lycopene and lutein) results in the modulation of the expression of not only heme-oxygenase 1 and matrix metallopeptidase 1 genes, but also of the intercellular adhesion molecule 1 [192].

### 8.4. Visible Light-Absorbing Coloured Carotenoids and Photoprotection in Humans

#### 8.4.1. Astaxanthin

Astaxanthin is a xanthophyll biosynthesized by microalgae (*Haematococcus pluvialis*, *Chlorella zofingiensis*, and *Chlorococcum* sp), the yeast *Phaffia rhodozyma* and the bacterium *Agrobacterium aurantiacum.* Animals (zooplankton, crustaceans, fish) incorporate it through the diet [193]. In general, the major dietary source for humans is salmon. Commercially, this carotenoid, which can be used as feed additive or human dietary supplement, is largely obtained by synthesis or biotechnologically from *H. pluvialis* [194]. There is evidence that astaxanthin could provide benefits at the level of skin, such as protection against erythema or reduced wrinkling [194]. 

#### 8.4.2. Canthaxanthin

Canthaxanthin, which is not one of the major dietary carotenoids but is approved as a food additive in many countries, has also proven useful to treat erythropoietic protoporphyria, although its accumulation in the retina over extended use raised concern about use of this carotenoid in general [175,195].

#### 8.4.3. Beta-Carotene 

Beta-carotene is a widely distributed carotenoid found in many foods. Some important sources are carrots, palm oil, mango, sweet potato, apricot, and green vegetables [15,72]. Beta-carotene has been long known to be beneficial for the treatment of erythropoietic protoporphyria, a rare inborn haematological disease that leads to increased levels of protoporphyrins in plasma, red blood cells, skin, and faeces. Protoporphyrin is an endogenous photosensitizer. This compound, upon exposure to UVR, becomes excited, and may eventually pass on the excitation energy to O_2_ in the ground state, thus generating singlet oxygen, which is a ROS that can interact with different molecules (DNA, proteins, lipids), causing cellular damage and leading to clinical symptoms of photosensitivity. Specifically, the patients experience itching, burning and pain in the skin exposed to sunlight after even only minutes of exposure, followed by edema, erythema and purpura [11,39,40,41,54]. The photoprotective effect of β-carotene has been the subject of several original research articles and reviews. Altogether, they indicate that it protects against erythema caused by UVR and that such protection requires daily dosages of about 10 mg for ~10 weeks [38,54,196,197,198,199]. On the other hand, a (9*Z*)-β-carotene-rich algal powder from *Dunaliella bardawil* has been shown to reduce the severity of psoriasis in adult patients with mild, chronic, plaque-type psoriasis as assessed by changes in Psoriasis Area and Severity Index (PASI) [200].

#### 8.4.4. Lutein

Lutein is a widely distributed dietary carotenoid present in all green vegetables, a wide variety of fruits, and egg yolk [72,75,201]. A recent placebo-controlled, double-blinded, randomized, crossover study has shown that an intervention with lutein capsules containing free lutein stabilized by 10% of the antioxidant carnosic acid, can protect against photodamage by decreasing the expression of UVR-modulated genes, including heme-oxygenase 1, intercellular adhesion molecule 1, and matrix metallopeptidase 1 genes [192].

#### 8.4.5. Lycopene

Lycopene is an acyclic carotenoid found in foods including some varieties of tomatoes, watermelons, guava, papaya, apricots, and grapefruits, together with other carotenoids, including the colourless phytoene and phytofluene [23,72,110,202].

Having been shown to be a quencher of singlet oxygen in vitro [203], several studies indicate that lycopene may provide photoprotection, although most of the in vivo studies have used products containing lycopene extracts (e.g., tomato paste in olive oil, diverse tomato extracts, or even a tomato-containing carrot juice) that also include other accompanying compounds (e.g., vitamin E or the colourless carotenoids phytoene and phytofluene) [204]. Being precursors of carotenoids and very close to lycopene in the biosynthetic path of carotenoids, phytoene and phytofluene are always found together with lycopene in the main sources of the latter, and also occur in other foods where lycopene is not found, such as carrots, citrus and others [23]. In this sense, the possible contribution of such components must be considered, in addition to the possibilities of interactions such as synergisms [50]. Indeed, some reviews indicate that there is little evidence of the health benefits of lycopene alone, since in most cases tomato extracts also containing other carotenoids are used [116,117]. Concerning photoprotection, this is well illustrated in an elegant study in which human volunteers received synthetic lycopene, a tomato extract or a tomato-based drink (all of them supplying virtually the same lycopene amount) for 12 weeks. The results indicated that the intervention resulted in the prevention of the UV-induced erythema formation in all groups, although the effect was more intense in the volunteers receiving tomato based products than in the group receiving synthetic lycopene alone, suggesting that phytofluene and phytoene may have contributed to the effect [113]. Recently, evidence became available that a lycopene-rich tomato product also containing phytoene and phytofluene, tocopherols, and phytosterols can provide photoprotection by inhibiting UVR-induced upregulation of heme-oxygenase 1, intercellular adhesion molecule 1 and matrix metallopeptidase 1 genes [192] 

Lastly it has been recently shown in SKH-1 hairless and immunocompetent mice that male mice that received diets containing lycopene, phytoene and phytofluene and other constituents from tomatoes developed fewer UVB-induced skin tumours relative to controls [205]. 

#### 8.4.6. Other Carotenoid-Containing Products

Recently, paprika oleoresin has been shown to be effective in humans in increasing MED and reducing UV-induced skin darkening. Paprika is a carotenoid-rich product obtained from red peppers containing red xanthophylls (capsanthin or capsorubin), as well as other xanthophylls (zeaxanthin and β-cryptoxanthin, among others) and carotenes (β-carotene, phytoene, and phytofluene) [72,110]. In a randomized, placebo-controlled, parallel-group comparative clinical study, daily oral supplementation with a red paprika product led to a significant enhancement of the MED and reduction of skin tanning in skin exposed to UV on the back, allegedly through antioxidant and anti-inflammatory mechanisms. At the end of the four-week study the treatment did not lead to statistically significant changes in a* (redness), transepidermal water loss (TEWL) or stratum corneum hydration (SCH) in the back area exposed to UVR, although the change in skin lightness was statistically significant, indicating that the intervention effects a change in skin colour after UV irradiation. To evaluate the effect of paprika xanthophylls on non-UV-irradiated skin, facial skin colour (L*, a*, b*), TEWL and SCH values were determined, although no significant differences were observed [206]. 

### 8.5. UV Light-Absorbing Colourless-Carotenoids (Phytoene and Phytofluene) and Photoprotection in Humans

One of the first studies pointing to the beneficial role of carotenoids in skin health concluded that injected doses of phytoene provided protection against UVR-induced erythema in guinea pigs [207]. The pioneer Mathews-Roth [208] described that the prolonged administration of phytoene to mice reduced the appearance of UV-B light-induced skin tumours and their multiplicity. Another major finding of this study was that no anticarcinogenic activity was noticed when the tumours were induced chemically. 

Regarding humans, aside from studies in which products containing lycopene plus colourless carotenoids were tested, there is further evidence that they exert photoprotection. Thus, a proprietary oral food supplement in the form of a tomato powder rich in PT and PTF (Israeli Biotechnology Research Ltd., Yavne, Israel) provided to women resulted in an mean increase of 10% in the minimum erythemal dose (MED), with 2/3 of the volunteers having a 20% increase at the end of the 12-week study [209].

#### Possible Mechanisms

Evidence is accumulating in relation to the beneficial effects of colourless carotenoids in terms of skin photoprotection, although the mechanistic aspects are still unclear. However, there is evidence of varying nature about actions that merit further investigation. 

As already commented, phytoene absorbs maximally in the UVB region (280–320) and phytofluene in the UVA region (320–400 nm), unlike virtually all other carotenoids (Table 2). Taking this into consideration, it is expected that these colourless carotenoids could provide photoprotection through the absorption of UVR [23].

Additionally, it has been reported that phytoene and phytofluene could protect against erythema and DNA damage caused by UVR and hydroxyl radicals and that they could have anti-inflammatory effects, as observed in human peripheral blood lymphocytes and in vivo in a mouse ear edema model [210]. Finally, in an in vitro study, human neonatal dermal fibroblast cell cultures were either subjected to UVR or exposed to interleukin-1 and then treated with coenzyme Q10, phytoene+phytofluene or combinations of the coenzyme and the carotenoids. The latter treatment was shown to lead to an enhanced anti-inflamatory response [122]. 

## 9. Carotenoids and Cosmetic Benefits

### 9.1. Carotenoids and Colour Signalling in Animals

The importance of the colour of the skin of animals or associated structures, e.g., feathers in birds, from several points of view (biological, social) is beyond doubt. Thus, carotenoid-based signals in animals advertise information about health/disease or nutritional status, genetic quality, aggressiveness, fertility, and so on [211]. For example, it is thought that male birds devoting higher levels of carotenoids to sexual-related colouration are communicating their better health [212]. Thus, according to the carotenoid trade-off hypothesis, using carotenoids for colouration prevents their use as antioxidants, such that the trade-off leads to a correlation between external colouration and health as healthy individuals can afford to use more carotenoids for colour. Contrastingly, according to the carotenoid protection hypothesis, carotenoid-based external colouration in animals could be a sign of the occurrence of other antioxidants that prevent carotenoids from being degraded by oxidation, with concomitant colour loss [59].

### 9.2. Carotenoids as Cosmetics

#### 9.2.1. From Cleopatra to Tanning Pills

Curiously, humans already used carotenoids topically with cosmetic purposes long before these compounds were “officially” discovered. Thus, it is reported that Cleopatra, the famous Pharaoh of Egypt, used saffron profusely for cosmetic purposes. Saffron is a product derived from the stigmas of *Crocus sativus* that exhibits a vivid orangish colour due to the presence of high levels of the apocarotenoid crocetin associated to sugar moieties. Similarly, indigenous people from South and Central America used to paint their faces with carotenoid-rich products, like annatto. This is obtained from the seeds of *Bixa orellana*, a small tree that bears in its scientific name the surname of the Spanish adventurer Francisco de Orellana, who is thought to have “discovered” it during his travels in the Amazonian region [213].

In modern times, carotenoids in supplements have been used since the 1970s as tanning agents, especially in Northern Europe. However, the use of canthaxanthin supplements raised concerns as it was observed that, in some cases, their continuous intake at high doses (from 30 mg/day) could lead to the formation of crystals of this carotenoid in the eye—though these could disappear when the intake of the product was discontinued [16,175]. Currently, EFSA recommends lower doses, specifically the Panel on Food Additives and Nutrient Sources added to Food (ANS) established an acceptable daily intake (ADI) of 0.03 mg/kg bw/day, which is in agreement with earlier recommendations made by the Joint FAO/WHO Expert Committee on Food Additives (JECFA) and the Scientific Committee on Food (SCF) [195].

#### 9.2.2. New Trends

Evidence that the intake of carotenoids from fruits and vegetables leads to perceived cosmetic benefits is accumulating in the last years. More specifically, the association between skin carotenoid-based colour and facial appeal has drawn attention only in the last years [59]. 

Dietary interventions with carotenoid-containing products leads to perceived changes in skin colour. Whitehead et al. [163] informed that self-reported increases in the dietary intakes of fruits and vegetables for 6 weeks led to increases of the CIELAB colour parameters a* and b* (regarded as estimations of redness and yellowness contributions to overall colour, respectively) in the skin. More recently, Pezdirc et al. [55] clearly demonstrated that the consumption of a diet high in fruits and vegetables by young women led to significant increases in the readings of the b* parameter in skin, consistent with increased yellowness.

In a very interesting paper, data on the contribution of melanin and carotenoids to the colouration of skin and the healthy appearance of human faces were evaluated [214]. It was concluded that the skin colour attributed to carotenoids is associated to the quality of the diet and health status and that such “carotenoid skin colouration” can be a valid cue of importance for the choice of mate [214], a situation similar to that described for other animals [211,212] Furthermore, the effect of facial skin carotenoid and melanin colouration on the perceived health was investigated. For this purpose, Caucasian participants were allowed to manipulate the skin colour of computer-generated facial images from the same racial group along carotenoid and melanin colour axes that were empirically measured. They were asked to “make the face as healthy as possible”. In this survey, it was concluded that the participants chose to enhance derived “carotenoid colouration” more than “melanin colouration” to maximize apparent facial health. This, according to the authors, indicated that the colouration imparted by carotenoids had more impact on the perceived human facial health than that attributable to melanin [214]. 

Evidence that skin colour linked to carotenoids is preferred over that attributable to melanin in certain populations is accumulating. Thus, in a recent report Lefevre et al. [215], provides more interesting information in this respect. The authors used controlled facial images manipulated to be high or low on carotenoid or melanin colouration to evaluate not only that aspect, but also whether both kinds of pigments had an impact on the perceived appeal and if the results were dependent on the gender of the face. The results of the study indicated that, under the experimental conditions, carotenoid colouration was consistently preferred over melanin with more marked preferences for carotenoids in female compared to male faces, regardless of the sex of the judging observer [215]. More recently, it has been concluded that young Australian adults see the skin colour of the face linked with carotenoids (derived from the consumption of fruit and vegetables) and melanin (owed to sunlight exposure) as conveying a healthy appearance in young adults, although carotenoid colouration was more important to health perception [216].

### 9.3. The Colourless UV-Absorbing Carotenoids Phytoene and Phytofluene in Cosmetics

Given their unique characteristics as colourless UV-absorbing carotenoids, phytoene and phytofluene offer distinctive possibilities relative to other carotenoids to provide cosmetic benefits at different levels.

#### 9.3.1. Skin Whitening by Phytoene and Phytofluene-Rich Products

An increase of the deposition of melanin in certain areas of the skin to produce dark spots and freckles is related to exposure to sun, skin disorders, aging or hormonal disorders. The importance of dark spots goes beyond aesthetic preferences since they may lead to cancer in some cases, hence the interest of using whitening agents [210]. The use of whitening agents for cosmetic and other purposes (psychological sociological, political or economic reasons) has been common in African and Asian societies, having been traced back to the 16th century in some Asian countries (India, China, Japan, or Korea). Currently, some commonly used whitening agents (corticosteroids, hydroquinone, monobenzyl hydroquinone, tretinoin, or mercury salts) raise concerns and are even forbidden in some countries due to associated side effects [217]. For example, hydroquinone at low levels (2%) has been the whitening treatment of choice for dyspigmentation, for decades, due to its ability of inhibit the activity of tyrosinase, the key enzyme for melanogenesis. However, the safety of its use raises controversies as it may cause irritation and has been associated to the possible development of malignancies [10]. In this context, the use of phytoene and phytofluene-containing products for skin whitening offers obvious advantages, including their safety, which may be assumed from their constant consumption in the human diet worldwide [29]. The use of these carotenoids for this purpose may also result in additional cosmetic and health benefits, due to their distinctive strong UV radiation absorbing properties and various attributed effects (antioxidant, antiinflammatory, anticarcinogenic [22,23,24].

Previous experiments with phytoene and phytofluene-rich topical formulations (IBR, Israeli Biotechnology Research Ltd., Yavne, Israel) result in skin lightening effects and beneficial effects like anti-ageing and anti-wrinkling effects [218,219]. More specifically, in a 12-week clinical study the skin whitening effect of a dietary supplementation with capsules providing 5 mg of phytoene and phytofluene per day) was evaluated in women with Fitzpatrick skin phototype IV (Table 1). In this study it was reported that a lightening effect, as assessed from increases in L* values and ITA, was observed in up to 82% of the volunteers [219]. L* is a measurement of the relative lightness of the skin, and places a given skin colour on the grey scale, between black (L* = 0) and white (L* = 100) [220], whereas ITA° is a parameter that helps assess the degree of skin pigmentation and is inversely related to skin lightness [209].

The study by von Oppen-Bezalel et al. [209] showed that, contrarily to the perceived change of skin colouration produced by other carotenoid-rich products [55,163], a tomato powder rich in colourless carotenoids (Israeli Biotechnology Research Ltd., Yavne, Israel) administered orally to women for 12 weeks at dietary achievable doses (~5 mg of PT plus PTF per day) increased MED but did not lead to statistically significant changes in the colour parameters L*, a*, and b* nor the individual typological angle (ITA°) Indeed, although the aim of the study was not to measure skin whitening (for example, women qualifying as skin phototype II were enrolled; see Table 1 for characteristics of this light phototype), the results obtained from the instrumental measurements carried out with a spectrocolourimeter revealed clear, albeit non-statistically significant, changes in L* [209] 

The results of these studies using phytoene and phytofluene-rich products open the doors to the oral use of these carotenoids in nutricosmetics as whitening agents [192], for the treatment of dark spots or to cater for the preferences of certain populations (like East Asians or Afro-Americans) for lighter skin colours. 

#### 9.3.2. Effect on Other Skin Aesthetical Parameters

The photoprotective effects of the intervention described in the study by von Oppen-Bezalel et al. [209] was accompanied by perceptible improvements in skin radiance, suppleness, evenness, smoothness, moisturization, elasticity, visible skin health, visible skin youthfulness, and overall skin beauty at the end of the 12-week study. Interestingly some enhancements were already noticed after 6 weeks. Notably, the improvements in the parameters were perceived both clinically and by the human volunteers [192,209]. Evidence of skin evening and anti-wrinkling effects derived from the use of topical formulations containing phytoene and phytofluene have also been reported [218,219].

Finally, the fact that phytofluene is fluorescent may have cosmetic implications and be harnessed for the development of innovative products.

### 9.4. Carotenoids and Aesthetic Benefits: Public Health Implications

It is well-known that the skin tone of certain ethnicities (for instance, Caucasians) is linked to the healthiness of the diet [8,214,221]. Overall, the results of some of the studies commented in previous sections, point to the fact that individuals, including young adults, from different geographical locations show preference for skin colouration linked to carotenoids (that is, to higher intakes of fruit and vegetables, their main dietary sources) over skin colouration associated with melanin (derived from sunlight exposure), which may be harnessed to increase fruits and vegetables intakes, especially in sectors with low intakes, like young adults [214,215,216]. Beyond skin tone, the intake of colourless carotenoid-containing products seem to have other aesthetical benefits at the level of the skin, resulting in the self-perception of the skin as more beautiful, healthier and younger [192,209]. On the other hand, a study involving young college women indicated that nutrition educators and health practitioners should be aware that such population groups (and probably many more similar groups) are less aware of and probably less interested in the health benefits derived from increasing fruits and vegetables intakes compared with older adults. Within this context, it seems sensible that such professionals revise their strategies and focus on aspects like showing to young men and women the relationship of fruit and vegetable intake with satiety, weight and appearance [222]. In this scenario, it is not surprising that the relationships between perceived attractiveness, skin pigmentation and the intake of carotenoid-rich fruits and vegetables could be used to boost the consumption of such products as a measure to tackle unhealthy dietary patterns leading to higher risks of several diseases, as envisaged by Whitehead et al. [223,224] who argued that this new paradigm based on an “appeal to vanity” in relation to carotenoid pigmentation can be an alternative to health-based messages. One example of this would be the recommendation of increasing the consumption of fruit and vegetables to reduce the risk of developing chronic diseases [225] which, according to Whitehead et al. needs alternatives, like interventions appealing to vanity, some of which have already proven promising [224].

Whitehead et al. [223] concluded that the majority of interventions targeting appearance stressed noncompliance-derived negative effects in attractiveness. For instance, exposure to young adults of UV photographs and photoaging information (for instance effect on wrinkles and age spots) appears promising as a brief and cheap strategy to increase young adults’ sun protection intentions and attitudes, which may in turn reduce their chances of developing skin cancer [226]. A new paradigm encouraging the intake of fruits and vegetables as a means to improve appearance seems promising, since showing individuals images of how their faces improve because of the dietary change is motivating. For this strategy to be viable, more studies providing empirical data about how the diet impacts the appearance are neede. For instance investigations comparing the current appearance of the individual and that achievable through better or worse dietary patterns. As already discussed, some studies indicate that individuals from different ethnicities are expected to prefer facial skin colouration attributable to increased fruit and vegetable intakes [214,221]. 

## 10. Conclusions

The protection of skin is important in the context of health as a means of preventing disorders that eventually lead to harmful conditions. Additionally, the appearance of the skin, notably the face, is an attribute of great relevance in signalling, since it conveys information with impacts at the socioeconomic level. 

The role of nutrition in skin health and appearance is undeniable and long-known, hence the efforts of the industry to innovate in cosmetics, cosmeceuticals, nutricosmetics, functional foods, or nutraceuticals. 

Carotenoids are natural dietary products that have been shown to intervene in health-promoting actions and whose value in the context of nutricosmetics continues to grow. From the literature it can be inferred that a diet rich in carotenoid-containing products and the avoidance of stress factors have a beneficial impact on skin health and appearance, with other likely beneficial systemic effects.

Excellent recent original studies and reviews point to the fact that the positive perceivable effects that dietary carotenoids cause in the skin may be harnessed in the context of public health. For instance, they can be used to promote healthy dietary patterns rich in carotenoid containing products as a strategy to reduce the risk of developing serious diseases, including cancer, cardiovascular disease, eye disorders, osteoporosis, or metabolic diseases. 

Among dietary carotenoids, the UVR-absorbing colourless carotenes phytoene and phytofluene have been largely overlooked, probably due to their lack of colour, which made their detection more challenging in the past compared to other carotenoids. Hence, the considerable lack of abundant data about their presence in foods and tissues, in contrast with the other major dietary carotenoids found in humans. However, it is well-established that they are major dietary carotenoids (found in products frequently consumed as tomatoes, carrots, citrus, and derivatives), present in plasma, human milk, skin, and other tissues, and involved in several health-promoting actions, as revealed by studies of different nature. Notably, evidence is accumulating that they could be involved in the health benefits traditionally associated to lycopene, since the latter seems to always occur along with the colourless carotenoids in foods. Being the unique major dietary carotenoids absorbing maximally in the UV region and possessing other distinctive characteristics within the carotenoid family, research and applications in the use of these carotenoids in the promotion of health and cosmetics is a timely and expanding area recently featuring in the carotenoid field. 

Future studies should devote attention to generating more data about phytoene and phytofluene skin levels in order to provide accurate information about skin carotenoid status, since mainly coloured carotenoids are currently being considered. Additionally, mechanistic studies about the beneficial effects of colourless carotenoids in the skin (whitening, improvement of aging signs, etc.) are needed. 

## Figures and Tables

**Table 1:** Characteristics of the different types of skin according to the Fitzpatrick–Pathak classification.

		Characteristics ^a^	
Skin type ^a^	Colour ^b^	Sunburn	Tan
I	White	Yes	No
II	White	Yes	Minimal
III	White	Yes	Yes
IV	White	No	Yes
V	Brown	No	Yes
VI	Black	No	Yes

^a^ Based on the responses to a verbal questionnaire related to the response of the skin to initial sun exposure, i.e., three minimum erythema doses (MEDs) or about 45–60 min of noon exposure in northern latitudes in the early summer. ^b^ Colour of the unexposed skin. From Fitzpatrick [35].

**Table 2:** Absorption maxima in acetone or petroleum ether, number of conjugated double bonds (c.d.b.) and colour of carotenoids important for skin health and appearance [99,100].

Carotenoid	Colour	c.d.b. (in rings)	Absorption maxima (nm)		
Canthaxanthin	Red	13 (4)		472	
Astaxanthin *	Red	13 (4)		468	
Lycopene	Red	11 (0)	446	474	504
β-Carotene	Orange	11 (2)		454	480
Zeaxanthin	Orange	11 (2)		454	480
Lutein	Yellow	10 (1)	424	448	476
Phytofluene *	Colourless	5 (0)	331	348	367
Phytoene *	Colourless	3 (0)		286	

* Absorption maxima in petroleum ether.

**Table 3:** Concentration of phytoene and phytofluene in body fluids (µM) and tissues (ng/g).

Body Fluid/ Tissue	Phytoene	Phytofluene	Reference
Blood	0.11 ± 0.01	0.30 ± 0.02	[111]
Blood	0.14 ± 0.08	0.14 ± 0.08	[112]
Blood	0.06 ± 0.04	0.33 ± 0.15	[113]
Blood	0.04	0.17	[114]
Breast	69	416	[115]
Cervix	-	106	[115]
Colon	70	116	[115]
Liver	168	261	[115]
Lung	1275	195	[115]
Milk	0.002	0.016	[114]
Prostate	45	201	[115]

**Table 4:** Carotenoid concentrations reported in human skin (nmol/g).

Carotenoid	Tissue	Concentration	Reference
α-Carotene	Abdominal skin	0.01	[154]
β-Carotene	Epidermis	0.39	[11]
β-Carotene	Dermis	0.01	[11]
β-Carotene	Epidermis	4.1	[11]
β-Carotene	Dermis	1.3	[11]
β-Carotene	Subcutis	3.5	[11]
β-Carotene	Surface lipid	10.0	[11]
β-Carotene	Comedones	14.5	[11]
β-Carotene	Whole skin	0.09	[11]
β-Carotene	Whole skin	1.41	[11]
β-Carotene	Punch biopsy	8.3	[11]
β-Carotene	Abdominal skin	0.05	[154]
γ-Carotene	Abdominal skin	0.04	[154]
ζ-Carotene	Abdominal skin	0.02	[154]
Lycopene	Abdominal skin	0.13	[154]
Phytoene	Abdominal skin	0.12	[154]
Phytoene	Whole skin	0.12	[115]
Phytofluene	Abdominal skin	0.03	[154]
Phytofluene	Whole skin	0.03	[115]
Total carotenoids	Whole skin	0.17	[113]
